# Enhanced transfection efficiency and targeted delivery of self-assembling h-R3-dendriplexes in EGFR-overexpressing tumor cells

**DOI:** 10.18632/oncotarget.4614

**Published:** 2015-07-20

**Authors:** Jun Li, Shengnan Li, Songyun Xia, Jinfeng Feng, Xuedi Zhang, Yanli Hao, Lei Chen, Xiaoning Zhang

**Affiliations:** ^1^ School of Medicine, Tsinghua University, Beijing 100084, China; ^2^ The third Clinical College, Southern Medical University, Guangzhou 510515, China; ^3^ Department of gynaecology and obstetrics, PLA Navy General Hospital, Beijing 100037, China; ^4^ Collaborative Innovation Center for Biotherapy, Tsinghua University, Beijing 100084, China

**Keywords:** h-R3, EGFR, gene therapy, PAMAM, targeted delivery

## Abstract

The efficient gene transfection, cellular uptake and targeted delivery *in vivo* are key issues for non-viral gene delivery vectors in cancer therapy. To solve these issues, we designed a new targeted gene delivery system based on epidermal growth factor receptor (EGFR) targeting strategy. An anti-EGFR monoclonal antibody h-R3 was introduced to dendriplexes of PAMAM and DNA via electrostatic interactions to form self-assembled h-R3-PAMAM-DNA complexes (h-R3-dendriplexes). Dendriplexes h-R3-dendriplexes represented excellent DNA encapsulation ability and formed unique nanostructures. Compared to dendriplexes, h-R3-dendriplexes presented lower cytotoxicity, higher gene transfection efficiency, excellent endosome escape ability and high nuclear accumulation in the EGFR-overexpressing HepG2 cells. Both *ex vivo* fluorescence imaging and confocal results of frozen section revealed that h-R3-dendriplexes showed higher targeted delivery and much better gene expression in the tumors than dendriplexes at the same N/P ratio, and h-R3-dendriplexes had accumulation primarily in the tumor and kidney. Moreover, h-R3-dendriplexes for p53 delivery indicated efficient cell growth inhibition and potentiated paclitaxel-induced cell death. These results indicate that the h-R3-dendriplexes represent a great potential to be used as efficient targeted gene delivery carriers in EGFR-overexpressing tumor cells.

## INTRODUCTION

The success of gene therapy for nonviral vectors depends on how efficiently the vectors can delivery therapeutic genes into target cells and high efficiency gene-expression *in vivo* [1–7]. The major approach in nonviral gene therapy is based on cationic polymers, which can mediate the delivery of DNA and RNA [8–14]. Among of theses polymers, polyamidoamine (PAMAM) offers such a nontoxic, nonimmunogenic and biocompatible gene carrier system. Many groups have used this polymer for gene delivery [15, 16]. Also, new PAMAM-derived modified polymers have been synthesized and evaluated *in vitro* by several authors, however, the efficiency of them is low and nonspecific *in vivo* [17, 18].

In order to improve selectivity and efficiency of the vector, nonviral systems have been conjugated with a variety of ligands, such as transferrin [19, 20], folate [21, 22], EGF [23] and antibody [24]. These modification ligands are mostly incorporated to the polymer vectors through chemical reactions, which are difficult to keep the bioactivity of the ligands [25, 26]. Thus, a better approach is needed to increase the transfection efficiency and maintain the bioactivity for the polymer mediated gene transfection at the same time. Compared to chemical modification, molecular self-assembly is a convenient strategy for making nano-complexes with remaining the bioactivity of the biomacromolecule [27].

Nimotuzumab (h-R3) is a humanized monoclonal antibody (mAb) that binds to the extracellular domain of the EGFR and inhibits EGF binding. H-R3 has been approved in several countries for the treatment of head and neck tumors, and is in clinical trials for various tumor types including cervical, colorectal, prostate, glioma, pancreatic, esophageal, and breast cancer [28–30]. Furthermore, one important advantage of using h-R3 in the clinic is the absence of severe adverse effects [31]. This makes the receptor as an attractive target for anticancer therapy.

With above-mentioned studies in mind, we investigate that the addition of the h-R3 to PAMAM mediated gene delivery system may increase the cellular uptake due to specific interactions between h-R3 and EGF receptors on tumor cells resulting in high transfection efficiency. To test this hypothesis, we prepared self-assembled h-R3-dendriplexes via electrostatic adsorption of PAMAM-DNA complexes to negatively charged antibody h-R3. Three different cell lines (EGFR-negative 293T, EGFR-expressing MCF-7 and EGFR-overexpressing HepG2) were used for *in vitro* experiments. The formulation, size, zeta potential, morphology and cytotoxicity of dendriplexes and h-R3-dendriplexes were evaluated by agarose gel retardation assay, dynamic light scattering, transmission electron microscopy and MTT assay. The *in vitro* gene transfection, cell uptake, *ex vivo* distribution and gene delivery were detected by flow cytometry, confocal laser scanning microscopy (CLSM), fluorescence imaging and confocal observation of frozen section. To test the potential of such novel gene delivery system in cancer gene therapy, we further investigated this h-R3-dendriplex system in p53 delivery against EGFR-overexpressing HepG2 and tested the efficacy.

## RESULTS AND DISCUSSION

### Formulation of h-R3-dendriplexes

**Figure 1:**
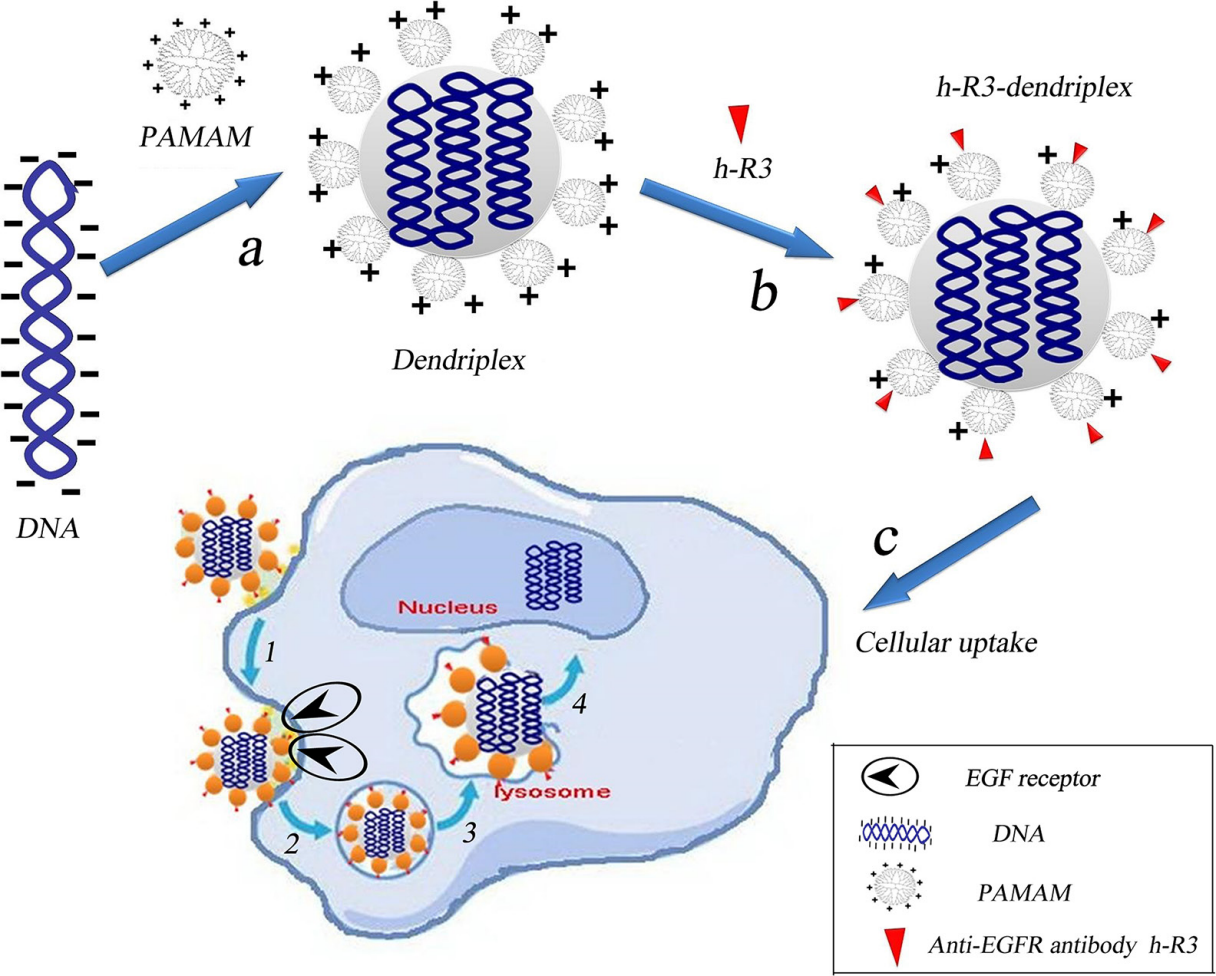
Schematic representation of the EGFR-based gene delivery system **a.** Electrostatic interactions of PAMAM and DNA to form dendriplexes. **b.** Self-assembled h-R3-dendriplexes via electrostatic adsorption of dendriplexes to negatively charged h-R3. **c.** h-R3-dendriplexes for targeted tumor gene therapy. 1, specific binding to the EGFR overexpressing receptors on the tumor cells; 2, receptor-mediated endocytosis; 3, captured by the lysosomes; 4, lysosomal escape and accumulation in the nucleus.

Amino-terminated PAMAM dendrimers with lower cytotoxicity have been extensively investigated as gene vectors. PAMAM dendrimers form complexes with DNA through electrostatic interactions between negatively charged phosphate groups of the nucleic acid and positively charged primary amino groups on the dendrimer surface. As we know, in order to condense DNA effectively, the dendriplexes for gene delivery usually have a positive charge on the surface [32, 33]. In the current study, the positively charged dendriplexes conjugated with the negatively charged anti-EGFR antibody h-R3 were designed. Figure 1 shows the schematic representation of the EGFR-based gene delivery system. Self-assembled h-R3-dendriplexes via electrostatic adsorption of PAMAM-DNA complexes to negatively charged h-R3 were designed. H-R3-dendriplexes can bind to the EGFR of EGFR positive tumor cell membrance. Then, the proton sponge effect caused by PAMAM dendrimer leads to lysosomal damage which can protect the DNA from the degradation in the lysosomes.

### Characterization of h-R3-dendriplexes

**Figure 2:**
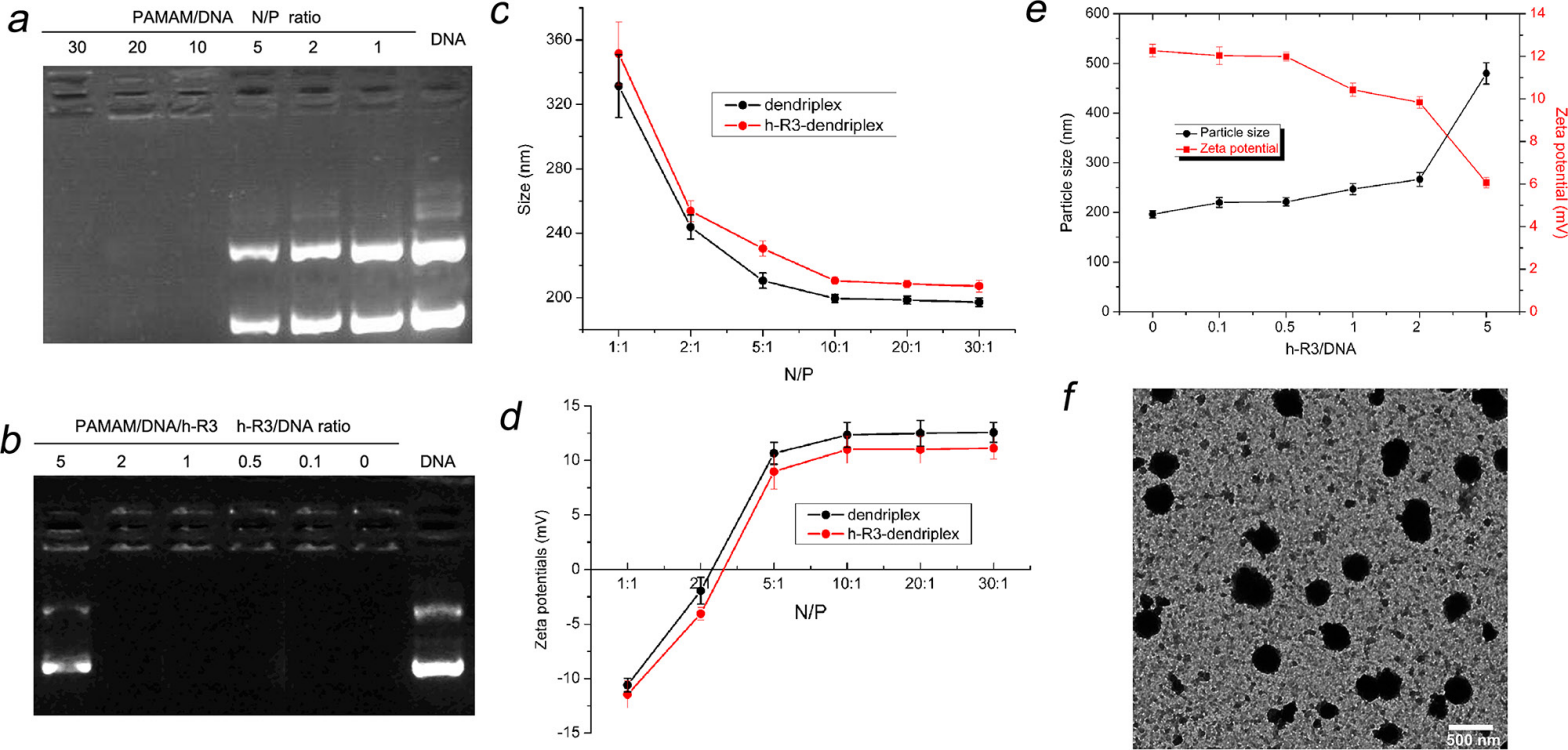
Characterization of dendriplexes and h-R3-dendriplexes **a.** Agarose gel retardation assay of dendriplexes at different N/P ratio (1, 2, 5, 10, 20, 30) **b.** Agarose gel retardation assay of h-R3-dendriplexes at different h-R3/DNA ratio (0, 0.1, 0.5, 1, 2, 5) at N/P of 20. **c.** Particle Sizes of dendriplexes and h-R3-dendriplexes at different N/P ratio. Results were expressed as mean ± standard deviation (*n* = 3). **d.** Zeta potentials of dendriplexes and h-R3-dendriplexes at different N/P ratio. Results were expressed as mean ± standard deviation (*n* = 3). **e.** Effect of h-R3 on the particle sizes and zeta potentials of h-R3-dendriplexes (N/P 20:1). **f.** TEM image of h-R3-dendriplexes (N/P 20:1 h-R3/DNA 1:1). Scale bar is 500 nm.

In this study, the formulation of dendriplexes and h-R3-dendriplexes with different N/P ratio and different h-R3/DNA ratio was also assessed by the agarose gel retardation assay and the results are shown in Figure 2a and Figure 2b. When the N/P ratios were reached 10, the dendriplexes were able to completely bind DNA (Figure 2a). However, for h-R3-dendriplexes with a N/P ratio of 20 (Figure 2b), h-R3-dendriplexes could not completely prevent DNA from migrating into the gel when the weight ratio of h-R3/DNA was 5, indicating that, in order to bind DNA completely, the weight ratio of h-R3/DNA should be less than 5. This result could be explained by a competition in the interaction between anionic DNA and negative antibody h-R3 with the positive PAMAM.

Dynamic light scattering (DLS) was employed in order to characterize the hydrodynamic size and zeta potentials of dendriplexes. The particle sizes of different complexes were measured as shown in Figure 2c. All sizes of dendriplexes at different N/P ratios were less than 250 nm, which indicated DNA could be effectively condensed by PAMAM at the N/P ratio higher than 10. As compared with the sizes of dendriplexes, the sizes of h-R3-dendriplexes at the same N/P ratio slightly increased. This is due to the fact that the negatively charged h-R3 was attached to the surface of dendriplexes, leading to the increased sizes. The particle sizes of nanoparticles have a significant impact on its delivery properties [34]. In order to investigate the effect of adding the targeting ligand on the sizes of dendriplexes, we compared the sizes of the h-R3-dendriplexes with increased h-R3/DNA ratio (Figure 2e). When the h-R3/DNA ratio increased, the sizes of h-R3-dendriplexes slightly increased.

A positive surface charge facilitates dendriplexes binding to the negatively charged cell surface, but excessive positive charge can lead to non-specific binding and significant toxicity [35]. The zeta potentials of the various complexes are shown in Figure 2d. The zeta potentials of dendriplexes were positive, when the excess PAMAM and the strong electrostatic interactions between dendriplex and h-R3 in the systems could form h-R3-dendriplex and the free h-R3 could be ignored. The zeta potentials of the dendriplexes increased with increasing N/P ratio when the N/P ratio was less than 10. Compared with dendriplex, the zeta potential of h-R3-dendriplex at the same N/P ratio decreased, because the negatively charged h-R3 was attached to the surface of dendriplexes. The zeta potentials of the h-R3-dendriplexes decreased when the h-R3/DNA ratio increased (Figure 2e).

The importance of particle shape on delivery properties is also gaining recognition [36]. Transmission electron microscopy (TEM) can provide direct information on the particle size, shape and morphology. Figure 2f shows the TEM images of h-R3-dendriplexes. The formulations confirmed spherical structures with mean particle size about 240 nm, which is a good agreement with the hydrodynamic size determined by DLS, as seen in Figure 2c. And, the size result partially met the enhanced permeability and retention (EPR) effect, which promotes nanoparticles to accumulate specifically in tumors [37, 38].

### *In vitro* transfection

**Figure 3:**
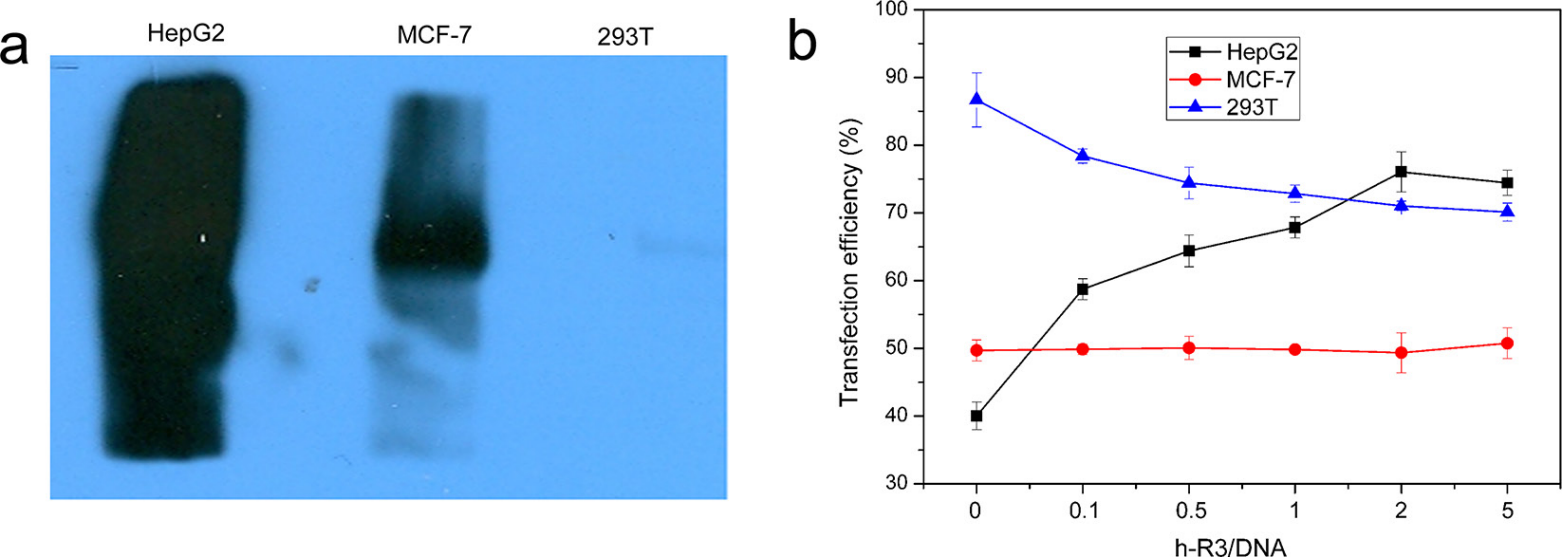
Different cell lines and Transfection efficiency **a.** Assessment of EGFR expression in different cell lines, including EGFR-overexpressing HepG2, EGFR-expressing MCF-7 and EGFR-negative 293T was determined by Western blotting. **b.** Transfection efficiency of h-R3-dendriplex with N/P 20:1, different h-R3/DNA (0, 0.1, 0.5, 1, 2 and 5) in HepG2, MCF-7 and 293T quantitatively by flow cytometer. Transfection was performed at a dose of 1 μg/well of pEGFP. Results were expressed as mean ± standard deviation (*n* = 3).

In this study, the transfection efficiency of dendriplexes was taken in EGFR negative cell line and EGFR-positive cell line. To assess the endogenous EGFR expression in the different cell lines, we detected EGFR protein level by western blotting. Among these cell lines, HepG2 cells have the highest level of EGFR expression; MCF-7 cells have moderate level of EGFR expression, whereas 293T cells have undetectable EGFR expression (Figure 3a). Based on the results, HepG2, MCF-7, and 293T cells were generated with high-, moderate-, and no-EGFR expression, respectively, for *in vitro* transfection studies.

Figure 3b showed the transfection efficiency of enhanced green fluorescent protein (EGFP) in different cell lines (EGFR-negative 293T cells, EGFR-expressing MCF-7 and EGFR-overexpressing HepG2) transfected with pEGFP-N1 by flow cytometry. In HepG2, the transfection efficiency obviously increased from 40% to 75% when the h-R3/DNA ratio increased from 0 to 5. And, the transfection efficiency kept almost the same about 50% on EGFR-expressing in MCF-7 and slightly decreased from 85% to 70% in 293T.

The effect of h-R3-dendriplexes on transfection efficiency could be attributed to zeta-potential and EGFR signal pathway. When h-R3/DNA weight ratio increased, the zeta-potential of h-R3-dendriplexes decreased (Figure 2e), which led to low transfection efficiency [39]. So, in EGFR-negative 293T, the reduced zeta-potential resulted in lowered transfection efficiency. And, on the other hand, EGFR signal pathway could promote clathrin-mediated endocytosis, which was effective to high transfection efficiency [40]. In EGFR-overexpressing HepG2, the EGFR signal pathway effect overcomes the disadvantage in reduced zeta-potential, and a significant increase in transfection efficiency was observed. The transfection efficiency of h-R3-dendriplexes in HepG2 is positively correlated with h-R3/DNA weight ratio. However, at extremely high h-R3/DNA weight ratios (h-R3/DNA = 5), transfection efficiency decreases. This results could be explained by the fact that the decreased DNA loading ability when h-R3/DNA weight ratios reached to 5 (Figure 2b), leading to low transfection efficiency. And, in EGFR-expressing MCF-7, the transfection efficiency of h-R3-dendriplex remained the same when the h-R3/DNA ratio increased. This may be attributed to the combination effect of reduced zeta-potential and EGFR signal pathway.

### *In vitro* cytotoxicity

**Figure 4:**
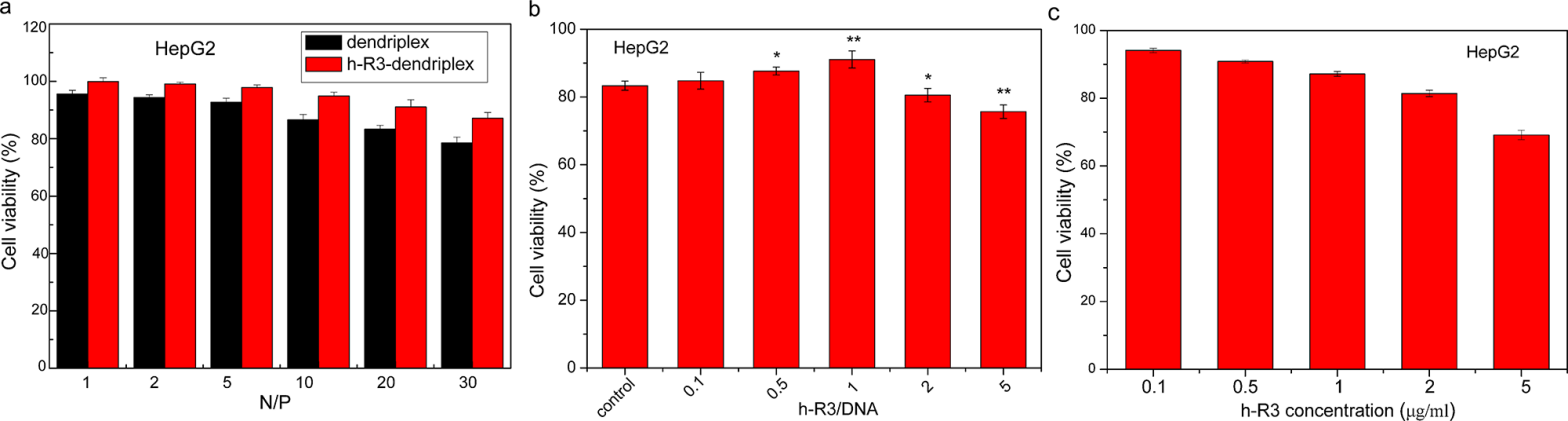
Cell viability valuated by MTT assay **a.** Cell viability after transfection mediated by different dendriplexes and h-R3-dendriplexes with different N/P ratios (1, 2, 5, 10, 20 and 30). The h-R3/DNA weight ratio is 1:1 for the h-R3-dendriplexes. Results were expressed as mean ± standard deviation (*n* = 3). **b.** Effect of h-R3 on cytotoxicity of h-R3-dendriplexes with increased h-R3/DNA ratio. Results were expressed as mean ± standard deviation (*n* = 3). **c.** Effect of h-R3 alone on cytotoxicity treated with HepG2 tumor cells (*n* = 3). *P < 0.05, ***P < 0.01 compared with the control group.

The cytotoxicity of different dendriplexes in EGFR-overexpressing HepG2 was evaluated by MTT assay. As seen in Figure 4a, the percentage of cell viability was reduced with the increase of N/P ratio in both dendriplexes and h-R3-dendriplexes. However, as compared to dendriplexes, the viability of treated with h-R3-dendriplexes was increased relatively at each N/P ratio. As we know, during the gene transfection, the positive charge on the surface of dendriplexes is essential for binding the negatively charged cell membranes and thus being taken up via an endocytic mechanism [41]. However, the higher positive surface charge means higher cytotoxicity of the dendriplexes, and the high cytotoxicity of the polycations limits their clinical applications as gene vectors. One of the strategies to reduce the cytotoxicity is to neutralize or partially shield the positive charge. In this study, h-R3 was used to partially neutralize the positive charge of dendriplexes. The h-R3-dendriplexes showed relatively lower zeta potential as compared with dendriplexes (Figure 2e), which resulted in their lowered cytotoxicity. Figure 4b showed the Effect of h-R3 on cytotoxicity of h-R3-dendriplexes with the increased h-R3/DNA ratio. The cell viability significantly decreased when the h-R3/DNA reached to 2 or 5, which can be explained by the fact that the present of h-R3 in h-R3-dendriplexes had the inhibition effect on the human hepatoma cell line HepG2, as shown in Figure 4c. These results revealed that the h-R3-dendriplexes also had the potentials for tumor therapy.

### Cellular uptake

**Figure 5:**
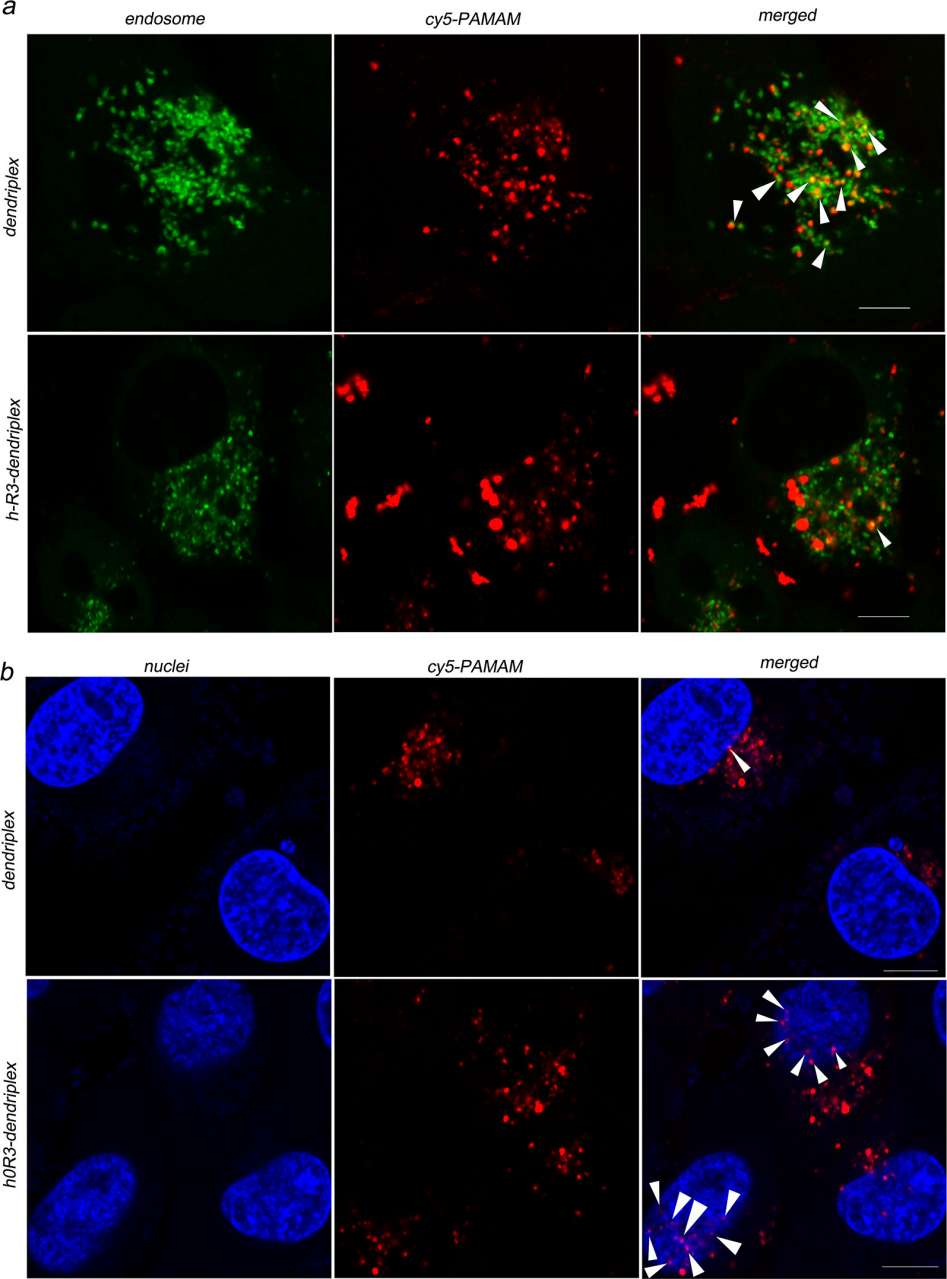
Cellular uptake observed by confocal laser scanning microscopy (CLSM) **a.** Internalization and subcellular localization of dendriplexes (N/P 20:1) and h-R3-dendriplexes (N/P 20:1, h-R3/DNA 1:1) in HepG2 cells. (green: LysoTracker Green used to label late endosomes and lysosomes; red: Cy5-labled PAMAM; yellow: colocalization). Scale bars = 5 μm. **b.** Intracellular localization of dendriplexes (N/P 20:1) and h-R3-dendriplexes (N/P 20:1, h-R3/DNA 1:1) in HepG2 cells. (red: Cy5-labled PAMAM; blue: Hoechst33342 stained cell nuclei; pink: colocalization). Scale bars = 5 μm.

The cellular uptake of dendriplexes and h-R3-dendriplexes was evaluated by CLSM after 24 h culturing with HepG2 cells. In this experiment, Cy5-labeled PAMAM were synthesized and employed to prepare dendriplexes. It is well known that the lysosomal escape of DNA and siRNA is crucial for the subsequent gene expression in the cytoplasm [42, 43]. To verify whether DNA escaped from the endosome/lysosome with the delivery of PAMAM-DNA, we incubated dendriplexes and h-R3-dendriplexes with HepG2 cells at 37°C, and examined the localization of DNA in cells by staining the acidic organelles (including endosomes and early lysosomes) with LysoTracker™ Green after 24 h of culture. As shown in Figure 5a, CLSM results indicated that after 24 h of incubation, the dendriplex was mainly colocalized with the LysoTracker™ Green stained organelles (arrows in Figure 5), suggesting that dendriplexes still resided in the endosomes or early lysosomes. However, it was clearly shown that incubation of cells with h-R3-dendriplexes resulted in separate localization of red and green fluorescence inside the cells, with minimal co-localization, which demonstrated that DNA appeared to have escaped from the lysosomal vesicles. Fewer obvious yellow regions and more red regions were observed in the cells treated with h-R3-dendriplexes compared to dendriplexes. It was indicated that more h-R3-dendriplex were distributed in the cells and released into cytoplasm while not in the endosomes. The results above may be due to the modification of h-R3, which facilitated the dendriplexes escape from the endosomes. Overall, the h-R3 conjugated dendriplex had excellent cellular uptake and endosome escape ability.

Nuclear translocation of the released DNA from the cytoplasm is crucial for effective gene delivery [44]. Figure 5b shows the intracellular localization of dendriplexes and h-R3-dendriplexes after 24 h of incubation in HepG2 cells. The cell nuclei were counterstained with Hoechst 33342. The results showed that incubation of cells with dendriplexes resulted in separate localization of red and blue fluorescence inside the cells, with few colocalizations (pink), and, h-R3-dendriplexes were mainly colocalized with the nuclei. Using the h-R3-dendriplex, DNA was transported into the nucleus with greater colocalization, indicating increased levels of translocation into the nucleus.

### *Ex vivo* distribution by fluorescence imaging and confocal microscopy

**Figure 6:**
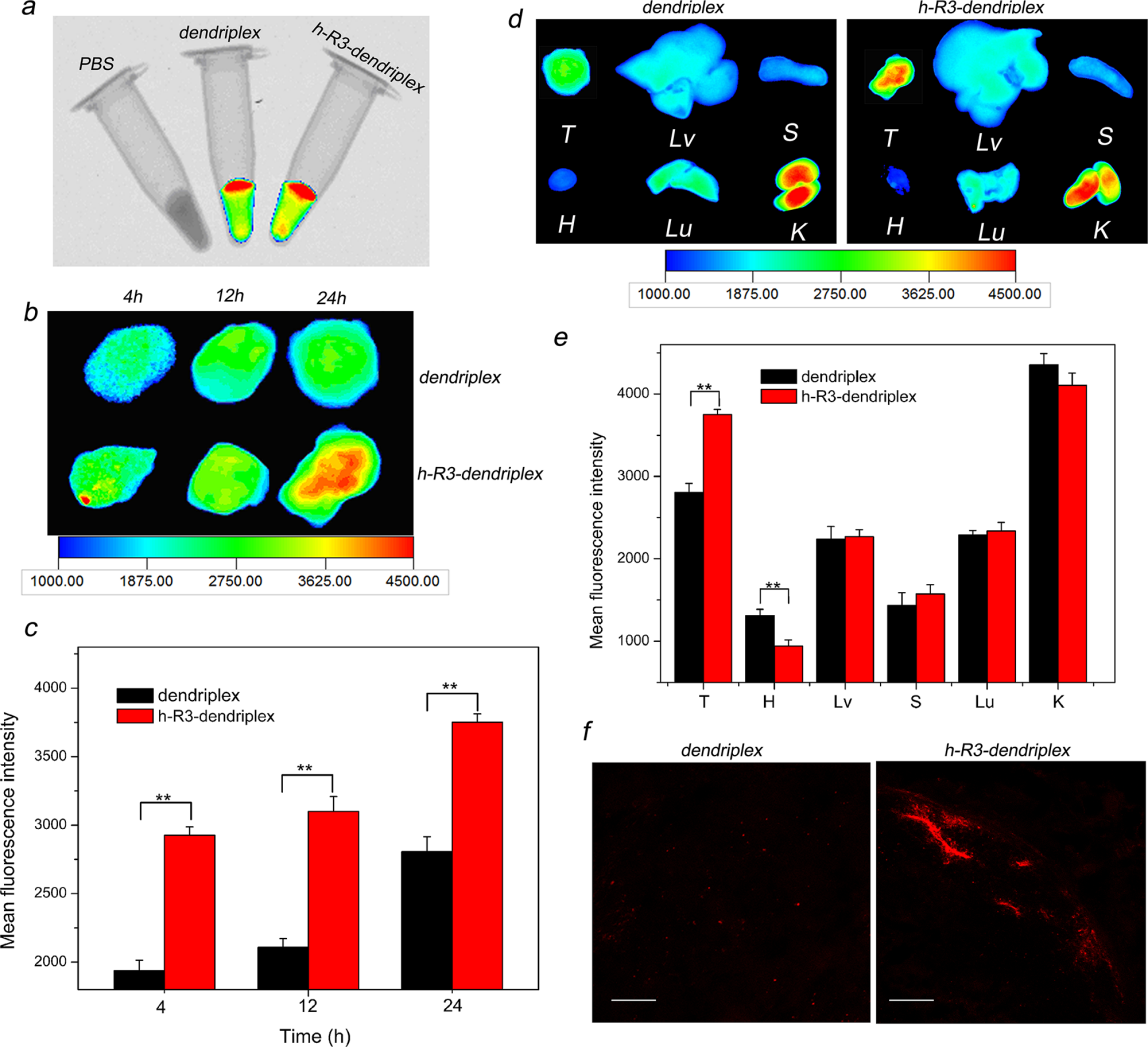
*Ex vivo* distribution and gene delivery of dendriplexes and h-R3-dendriplexes **a.** Overlay images of X-ray and fluorescence imaging of three samples *in vitro*. **b.** *Ex vivo* fluorescence imaging of the tumor at 4 h, 12 h and 24 h after i.v. injection to HepG2 tumor-bearing nude mice. **c.** Region-of-interest analysis of fluorescent signals from the tumors 4 h, 12 h and 24 h after i.v. injection to mice. Error bars indicate s.d. (*n* = 3). **P < 0.01. **d.** *Ex vivo* fluorescence imaging of five major organs and tumor 24 h post-injection (T, tumor; H, heart; Lv, liver; S, spleen; Lu, lung; K, kidney). **e.** Region-of-interest analysis of fluorescent signals from the tumors and normal organs. (T, tumor; H, heart; Lv, liver; S, spleen; Lu, lung; K, kidney). Error bars indicate s.d. (*n* = 3). ***P < 0.01. **f.** *Ex vivo* distribution of gene expression in the tumor 24 h after i.v. injection to nude mice-bearing HepG2 tumor. Frozen tumor sections (6 um thick) were examined by CLSM. Red homogeneous spots present the Cy5-dendriplexes and Cy5-h-R3-dendriplexes. Scale bars = 100 μm.

Fluorescence measurements allow a direct and comparative study of the biofate of nanoparticles. In this study, Cy5-labeled dendriplex and h-R3-dendriplex were used for fluorescence imaging to determine the biodistribution, and the PBS samples as a control. As seen in Figure 6a, the *in vitro* fluorescence intensities of dendriplex and h-R3-dendriplex keep the same, and can be available for *ex vivo* fluorescence imaging. To analyze dendriplex accumulations in organs which were not available by *in vivo* fluorescence imaging, *ex vivo* studies were usually made to get more detailed information. Figure 6b shows the *ex vivo* imaging results and fluorescence intensities of excised tumors injection after different times (4 h, 12 h and 24 h). For dendriplex group, low fluorescence signal were found in first 4 h after i.v. injection, and the visible accumulation in tumors remained more or less constant in 24 h. For h-R3-dendriplex group, fluorescence signal in tumors remained more or less constant during the first 12 h, and an increased signal was observed at 24 h. To determine the more detailed information on the fluorescence intensities of tumors, region-of-interest analysis of fluorescent signals from the excised tumors was presented in Figure 6c. Compared to the dendriplex, the *ex vivo* distribution results of h-R3-dendriplex revealed a higher dye intensities in the tumor at the same time point (4 h, 12 h and 24 h). These results revealed that h-R3-dendriplexes had better gene delivery efficiency and high targeted delivery than dendriplexes.

As seen in Figure 6d and Figure 6e, *ex vivo* imaging results and fluorescence intensities of five major organs and tumor 24 h after i.v. injection were presented. H-R3-dendriplexes showed accumulation primarily in the tumor and kidney, with only little fluorescence intensity in liver, spleen, lung and heart. These results seem very interesting for tumor-targeted gene delivery; while nanoparticles were mostly prefer to accumulate in liver and spleen due to the mononuclear phagocyte system [45]. The Anderson group also presented the similar results that molecularly self-assembled nucleic acid nanoparticles accumulated primarily in the tumor and kidney at 12 h post-injection by *ex vivo* fluorescence image [4]. The accumulation of dendriplexes in the kidneys indicated that they were excreted with the urine. In addition, the fluorescence signal at the tumor site was approximately double than that in the liver or lung 24 h after injection of h-R3-dendriplexes (Figure 6e).

To further evaluate the differences between the dendriplexes and h-R3-dendriplexes, we used CLSM to observe the complexes distribution in tumor. As shown in Figure 6f, h-R3-dendriplexes exhibited stronger fluorescence signals than dendriplexes group. These CLSM results are in accordance with the *ex vivo* results in Figure 6b and Figure 6c. Therefore, the more notable signals of Cy5 in h-R3-dendriplexes were probably due to the enhanced cellular uptake and endosome escape ability as discussed in Figure 5a.

### H-R3-dendriplexes for p53 delivery

**Figure 7:**
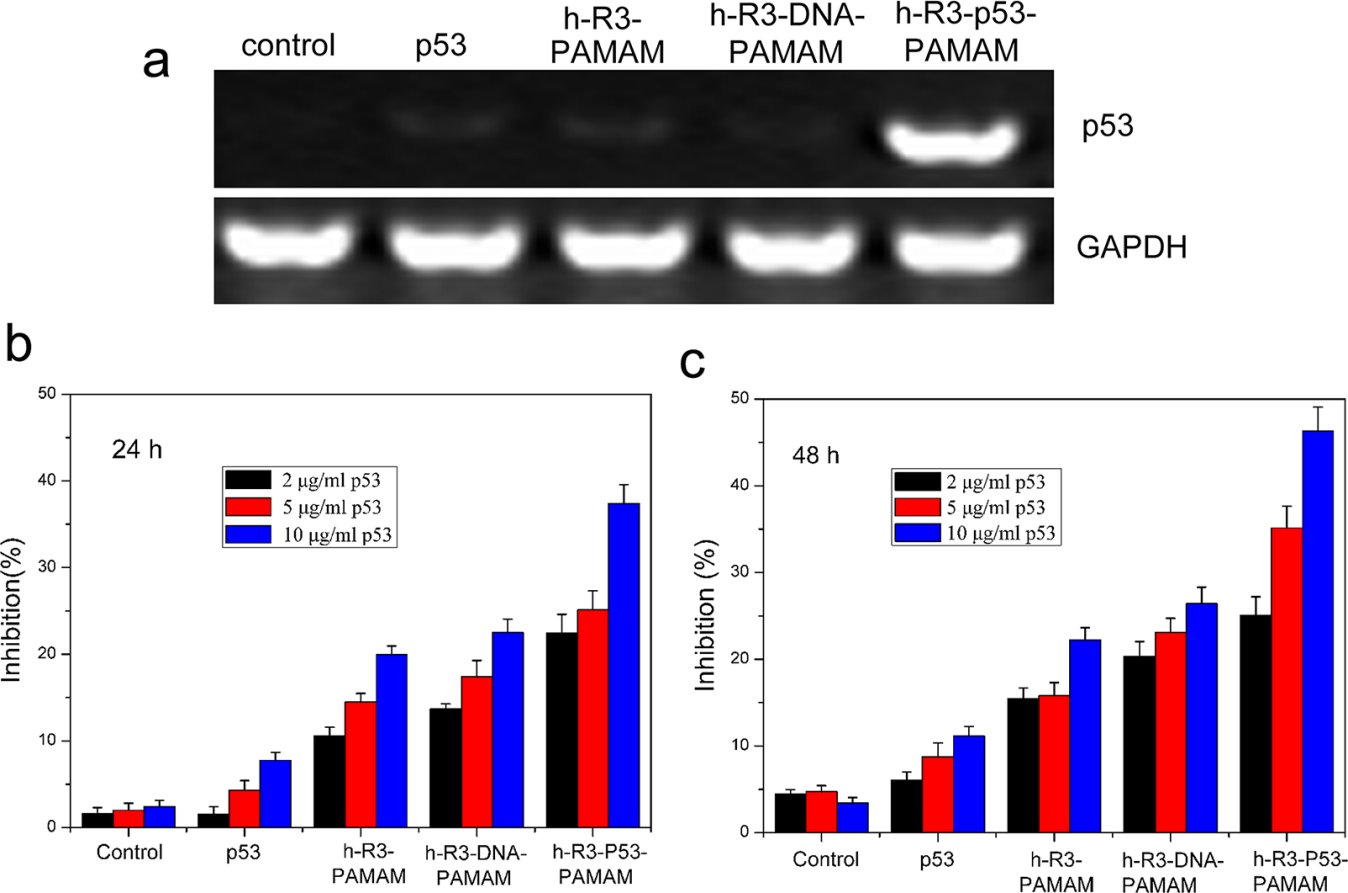
H-R3-dendriplexes for p53 delivery **a.** Expression of p53 at mRNA after transfection with different samples (naked p53, h-R3-PAMAM, h-R3-DNA-PAMAM and h-R3-p53-PAMAM) in HepG2 cells by RT-PCR; **b.** Cell growth inhibition induced by different p53 concentration treated after 24 h (2 μg/ml, 5 μg/ml, 10 μg/ml). N/P 20:1, h-R3/p53 1:1. Results were expressed as mean ± standard deviation (*n* = 3). **c.** Cell growth inhibition induced by different p53 concentration treated after 48 h (2 μg/ml, 5 μg/ml, 10 μg/ml). N/P 20:1, h-R3/p53 1:1. Results were expressed as mean ± standard deviation (*n* = 3).

As an important tumor suppressor gene, p53 is crucial for the regulation of normal cellular activities, including cell cycle, proliferation and apoptosis [46], and is found mutated or inactivated in most human tumors [47]. Delivering p53 effectively is most important to tumor therapy. As shown in Figure 7a, expression of p53 at mRNA in HepG2 cells was detected by RT-PCR. The results represented appreciable alterations in p53 mRNA levels after transfection with h-R3-p53-PAMAM compared to naked p53, h-R3-PAMAM and h-R3-DNA-PAMAM samples. Expression of GAPDH was used as an internal control to demonstrate equal loading of RNA samples. To evaluate cell proliferation, we used MTT assays (Figure 7b and Figure 7c). An efficient inhibition of cell proliferation was observed after treatment with h-R3-p53-PAMAM complexes for 6 h and subsequent incubation of up to 24 h or 48 h, relative to the naked p53 plasmid, h-R3-PAMAM, h-R3-DNA-PAMAM complexes as well as untreated sample. And, the result also showed that the increased p53 concentration led to high cell inhibition.

### H-R3-p53-PAMAM-complexes potentiates paclitaxel-induced death

**Figure 8:**
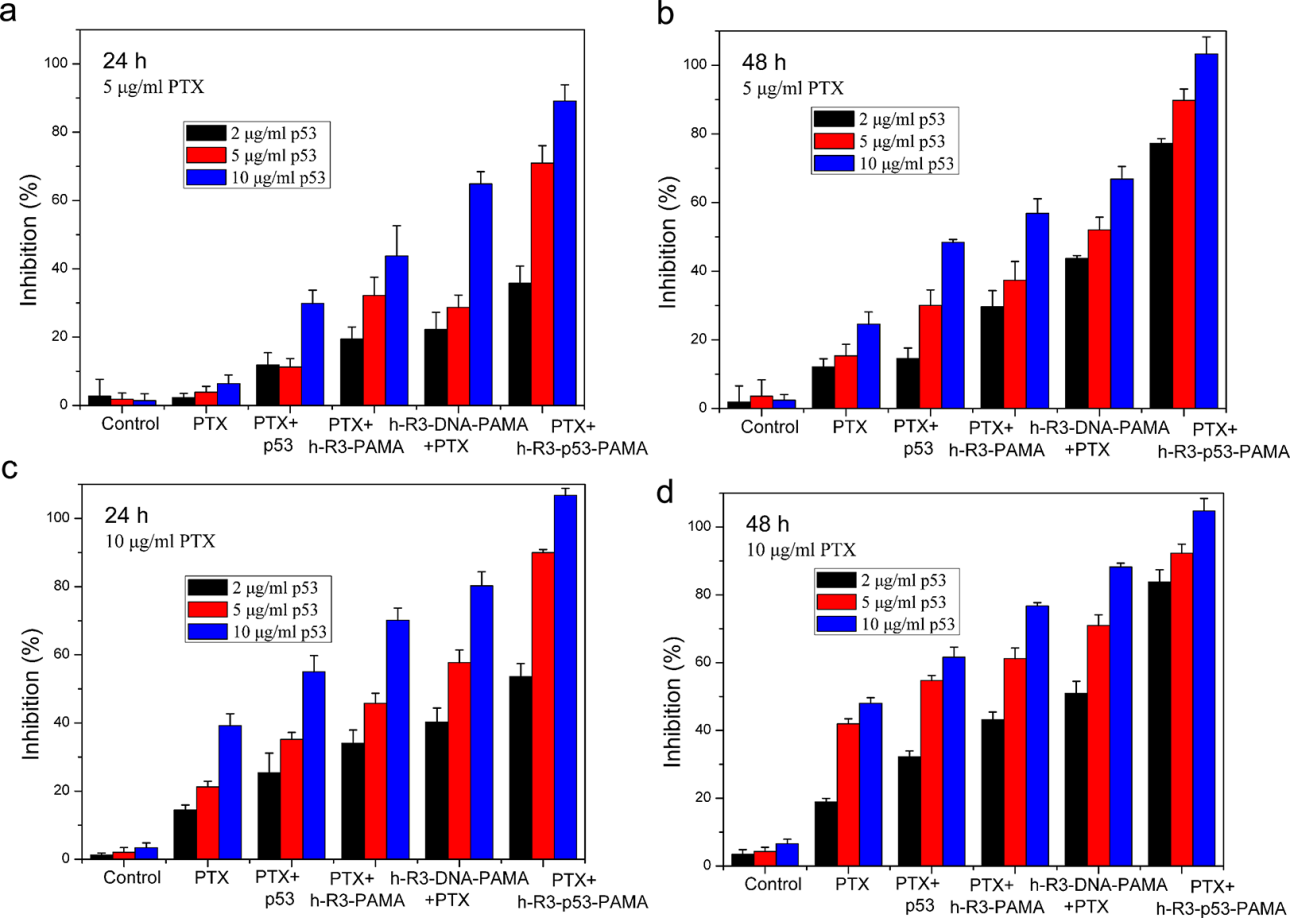
Cell growth inhibition induced by the combination of p53 and PTX treated after 24 h or 48 h **a.** 24 h, 5 μg/ml PTX; **b.** 48 h, 5 μg/ml PTX; **c.** 24 h, 10 μg/ml PTX; **d.** 48 h, 10 μg/ml PTX. N/P 20:1, h-R3/p53 1:1. Results were expressed as mean ± standard deviation (*n* = 3).

Paclitaxel (PTX) is the first-line chemotherapeutic drug used for treating cancer in the clinic. PTX blocks mitosis by impairing spindle function [48], a tumor-suppressing mechanism totally different from that of p53, and the interaction between PTX and p53 remains to be clarified. Figure 8 shows the cell growth inhibition induced by the combination of p53 and PTX. Upon combined treatment of h-R3-p53-PAMAM complexes and paclitaxel, we found a significant inhibition of growth of HepG2 cells. Our results provided evidence that p53 gene transfected with h-R3-dendriplexes could enable better effect of low-concentration PTX, which can be a potential auxiliary cancer therapy.

**Figure 9:**
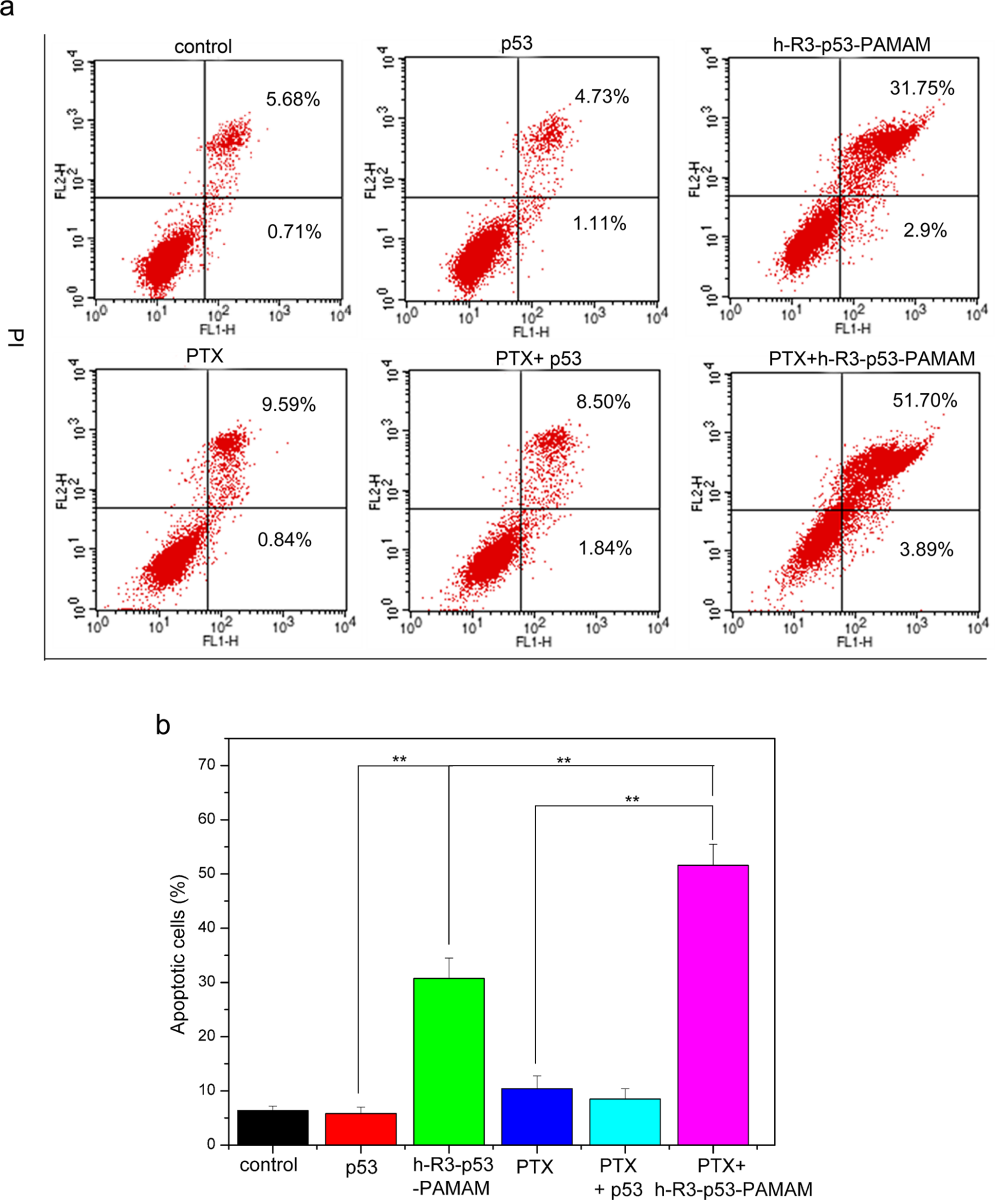
FACS analysis for detection of apoptotic cells **a.** FACS dot plots of HepG2 cells with different samples (p53, h-R3-p53-PAMAM, PTX, PTX+p53, PTX+h-R3-p53-PAMAM); **b.** Quantitative presentation of DNA fragmentation data from FACS analysis to indicate percent changes in apoptotic cells. Results were expressed as mean ± standard deviation (*n* = 3). **P < 0.01.

To assess whether apoptosis accounted for the loss of viability, we examined the expression by FACS analysis. An increase in cell population in the apoptotic cells area was shown in the FACS dot plot graphs (Figure 9a). We presented the quantitative FACS data for percentage of apoptotic cells based on DNA fragmentation (Figure 9b). Quantitation of data demonstrated 5.04%, 30.75%, 9.83%, 8.55% and 51.59% apoptotic cells after treatment with naked p53, h-R3-p53-PAMAM, PTX, combination of PTX and naked p53, and combination of h-R3-p53-PAMAM and PTX, respectively (Figure 9b). Results showed that h-R3-p53-PAMAM had higher apoptotic cells than p53, and indicated the treatment with combination of PTX and h-R3-p53-PAMAM caused more apoptosis in HepG2 cells than either treatment alone. The concomitant with an increase in PTX-induced cell apoptosis compared to treatment with either h-R3-p53-PAMAM complexes alone or PTX alone made cause to cell inhibition (Figure 8).

## MATERIALS AND METHODS

### Materials

Poly(amidoamine) (PAMAM) dendrimer with a ethylenediamine core (generation 5 with 128 surface amino groups) was purchased from Sigma (Shanghai, China). Nimotuzumab (h-R3) was a gift from BioTech Pharmaceuticals Co., Ltd. (Beijing, China). The reporter plasmid, pEGFP-N1, was purchased from Invitrogen (Carlsbad, CA, USA). 3-(4, 5-Dimethylthiazol-2-yl)-2, 5-diphenyltetrazolium bromide (MTT) and dimethylsulfoxide (DMSO) were from Sigma (Shanghai, China). The fluorescent dyes (Cy5 and Hoechst 33342) were purchase from Fanbo Biochemical Co. (Beijing, China). Annexin V-FITC/PI Apoptosis Detection Kit was got from Solarbio LIFE SCIENCES (Beijing, China). The plasmid pFLAG-CMV2-p53 encoding p53 was a gift from Prof. Sun of Tsinghua University, recombined from pFLAG-CMV2 and wild-type human p53 cDNA. Paclitaxel injections were purchased from Haikou Pharmaceutical Factory Co., Ltd (Haikou, China). All other reagents were obtained from the Biodee Reagent Company (Beijing, China).

### Cell culture

293T, MCF-7 and HepG2 cells were obtained from China Center for Typical Culture Collection (Beijing, China) and cultured in Dulbecco's Modified Eagle's Medium (DMEM) (Gibco), supplemented with 10% fetal bovine serum (FBS) and 100 unit/mL penicillin/streptomycin. All cell lines were cultivated in a humidified atmosphere of 5% CO_2_ at 37°C.

### Preparation of dendriplexes and h-R3-dendriplexes

PAMAM was diluted to an appropriate concentration in PBS and stored at 4°C until use. PAMAM/DNA complexes (dendriplexes) were made up by adding the aqueous solution of plasmid DNA to an equal volume of PAMAM in PBS at a particular N/P ratio, followed by incubation for 20 min. The N/P ratio was based on the calculation of the number of terminal -NH2 groups on the dendrimer versus the number of phosphate groups of the DNA. H-R3-dendriplexes were prepared by dropping the dendriplex aqueous solution to an aqueous solution of h-R3 of equal volume and then incubating for 20 min.

### Size distribution and zeta potential measurements

Size distribution and zeta potential of the dendriplexes and h-R3-dendriplexes with different N/P ratio and h-R3/DNA ratio were measured using a Zetasizer Nano ZS90 (Malvern Instruments Ltd, Malvern, UK). Measurements were performed at a temperature of 25°C after equilibration for 2 min. All results were the mean of three test runs.

### Agarose gel electrophoresis

Dendriplexes and h-R3-dendriplexes with different N/P ratio and h-R3/DNA ratio were evaluated by agarose gel retardation assay. Twenty microliters of complexes containing solution with 1 μg DNA was electrophoresed on the 1% (w/v) agarose gel containing ethidium bromide with Tris-acetate-EDTA (TAE) running buffer at 110 V for 30 min. DNA was visualized on a Vilber Lourmat UV transilluminator.

### Transmission electron microscope (TEM)

The dried specimens were examined with a Zeiss EM 900 transmission electron microscope at an acceleration voltage of 80 kV. Electron micrographs were taken with a slow scan camera (Variospeed SSCCD camera SM-1k-120, TRS, Moorenweis, Germany).

### Western bloting

Collecting the same amount of the different cells (293T, HepG2 and MCF-7), cells were washed twice with ice-cold PBS, homogenized on ice for 30 minutes in 10 volumes lysis buffer containing 20 mM Tris-HCl, 1 mM EDTA, 50 mM NaCl, 50 mM NaF, 1 mM Na3VO4, 1% Triton-X100 and 1 mM PMSF. The homogenate was centrifuged at 15, 000 rpm for 30 minutes at 4°C. The supernatant was collected and stored at −20°C. Protein content was determined by the NanoDrop 2000c. From each sample preparation, 50 μg of total protein was separated by 8% SDS-PAGE and then transferred to PVDF blotting membranes. The total protein extracts were analyzed by immunoblotting with indicated antibodies following SDS-PAGE analysis. Immunoblots were performed using rabbit monoclonal primary antibodies specific for EGFR (1:10000, Abcam). After blocking nonspecific binding with 5% BSA in TBS (pH 7.5) containing 0.05% Tween-20 (TBST), primary antibodies were incubated on the membranes for EGFR overnight at 4°C in TBST. Following five times washes in TBST, each time for five minutes, and the membranes were incubated for 1 h at 37°C with secondary goat anti-rabbit IgG antibodies (1:7500). Specific bands for EGFR were identified by prestained protein molecular weight marker.

### *In vitro* transfections

For transfection, 50000 cells were seeded in 1 ml of medium in 24-well culture plates and incubated for overnight at 37°C in 5% CO2. After this, the medium was removed and dendriplex/h-R3-dendriplex opti-MEM solution (200 μl containing 1 μg DNA) was added to each well. After incubation for 5 hours, the dendriplex was replaced and the cells were further incubated for 48 hours in medium containing 10% FBS. After transfection, cells were detached by trypsin. Cell suspensions were then transferred to microtubes and fixed by 0.2 mM EDTA. The percentage of cells transfected was quantitatively by flow cytometry using fluorescence-activated cell sorting (FACS) machine. Transfection efficiency was calculated based on the percentage of the cells that expressed pEGFP (positive cells) in the total number of cells.

### *In vitro* cytotoxicity

The cytotoxicities of dendriplexes and h-R3-dendriplexes with different formulations were examined by MTT assay. HepG2 cells were seeded in 96-well plates at a density of 10000 cells/well in 200 μl of DMEM containing 10% FBS. After incubation for overnight, the dendriplex/h-R3-dendriplex opti-MEM solution (20 μl containing 0.2 μg DNA) was added into each well. After 4 h, 200 μl fresh complete DMEM was added, and transfection proceeded for an additional 44 h in the presence of 10% FBS. Then, 20 μl of MTT (5 mg/ml) solution was added to each well, and further incubated for 4 h. Thereafter, the medium was carefully removed and 150 μl DMSO was added to each well to dissolve the formazan crystals. The absorbance was measured at 490 nm by a microplate reader. The cells without co-incubation with the dendriplexes were used as the control.

### Confocal laser scanning microscopy (CLSM)

PAMAM was labeled with Cy5 mono-reactive NHS ester (Fanbo Biochemicals, Beijing, China). The labeling reaction and purification of Cy5 labeled PAMAM were according to the manufacturer's protocol. HepG2 cells were plated at 5 × 10^4^ cells/well 12 h before transfection. After the culture medium was changed to opti-MEM, well incubated dendriplexes (N/P 20:1) and h-R3-dendriplexes (N/P 20:1, h-R3/DNA 1) solution containing 2 μg DNA were added into each plate. After 4 h incubation at 37°C in 5% CO_2_ humidified atmosphere, the transfection solutions were aspirated and substituted with complete culture medium. After additional 20 h incubation, the cells were washed with PBS for three times before acidic late endosome staining with LysoTracker green (Life Technologies). For confocal laser scanning microscopic measurements, a laser scanning microscope LSM 710 with Plan-Apochromat 100x/1.40 Oil DIC M27 objective (Zeiss, Germany) was used.

### *Ex vivo* distribution

All procedures of the *ex vivo* experiments complied with the standards for use of animal subjects as stated in the guidelines from Committee on Animal Research in Tsinghua University. The *ex vivo* studies were performed in female BALB/c nude mice from the Experimental Animal Center (Tsinghua University, Beijing, China). They were housed under controlled conditions (12 h light/dark schedule, 24°C) in groups of three mice per cage and formulation. Tumors were introduced in the armpits of the nude mice by inoculation with 10^7^ HepG2 cells. Palpable subcutaneous tumors developed over a period of 14 days. To monitor dendriplexes and h-R3-dendriplexes *ex vivo*, PAMAM was labeled with Cy5 (Fanbo Biochemicals, Beijing, China). Distribution was assessed by injecting Cy5-labeled dendriplexes (N/P 20:1) and Cy5-labeled h-R3-dendriplexes (N/P 20:1, h-R3/DNA 1:1) into tumor-bearing mice via the tail vein in a total volume of 200 μl (containing 10 μg DNA per mouse). The mice were sacrificed and organs were subjected for *ex vivo* imaging by the Kodak *in vivo* imaging system FX-Pro. The optical imaging was obtained using Kodak multimodal imaging system FX-Pro equipped with an excitation bandpass filter at 630 nm and an emission at 700 nm. Region-of-interests were circled around the organs, and the fluorescence intensities were analysed with the Carestream MI SE 5.4.2 software package.

### Frozen section preparation and *ex vivo* confocal observation

The tumor tissues were placed on omnisette tissue cassettes and rapidly frozen to −20°C. The specimens were embedded in optimal cutting temperature (OCT) compound, and cut into 6 mm histology slices by the cryostat. Each section was picked up on glass slides, and then covered with coverslip. Finally, frozen sections were observed using a confocal microscope (LSM 710, Carl Zeiss, Germany).

### RT-PCR for p53 mRNA detection

HepG2 cells were plated in a 6-well plates (4 × 10^5^ cells per well) and transfected with different samples (naked p53, h-R3-PAMAM, h-R3-DNA-PAMAM and h-R3-p53-PAMAM) for 48 h. Total RNA was isolated with TransZolTM Up (TransGen Biotech, Beijing, China). The reverse transcription reaction was performed using TransScript First-Strand cDNA Synthesis SuperMix System (TransGen Biotech, Beijing, China). For detection of p53 transcript, 3 μl cDNA was used in 20 μl reaction with p53 primers (forward, 5-CTACAAGCAGTCACAGCACATGAC-3; reverse, 5-TCATTCAGCTCTCGGAACATCTCG-3). GAPDH primers (forward, 5-GCCAAAAGGGTCATCATCTC-3; reverse, 5-GTAGAGGCAGGGATGATGTTC-3) were used as a control. The cycling procedure was as follows: 94°C for 5 min, then 30 cycles at 95°C for 30 s, 58°C for 30 s, 72°C for 60 s, followed by 72°C for 5 min. A volume of 20 μl PCR reaction was electrophoresed on a 1% agarose gel, and the amplified DNA band was visualized by DuRed (Bridgen Biotech, Beijing, China) staining.

### MTT assay for studies of cell growth curve and therapy efficacy

HepG2 Cells were seeded in 96-well plates at a density of 10000 cells/well in 200 μl of DMEM containing 10% FBS. After incubation for overnight, the samples with different p53 concentration (2 μg/ml, 5 μg/ml, 10 μg/ml) were added into each well for transfection. After 6 h, 200 μl fresh complete DMEM was added (for combined medication study, 5 μg/ml or 10 μg/ml paclitaxel was added together), and transfection proceeded for an additional 18 h or 42 h in the presence of 10% FBS. Then, the MTT detection method was described as Part 2.9.

### Apoptosis analysis by annexin V-FITC and propidium iodide (PI) double staining

HepG2 cells were plated in a 6-well plates (4 × 10^5^ cells per well) and transfected as described above. After incubation with treated samples for 48 h, the treated cells were washed, trypsinized and centrifuged. The cells were collected and re-suspended in 200 μl of Binding buffer, and 10 μl Annexin V-FITC and 10 μl PI (Solarbio LIFE SCIENCES, Beijing, China) were added. Furthermore, the stained cells were incubated at room temperature for 15 min in the dark, and analyzed by FACSCalibur flow cytometer with WinMDI 2.9 software.

### Statistical analysis

Statistical analysis was performed for statistical significance using the two-tailed Student's *t*-test. A value of *P* < 0.05 was considered as statistically significant, and *P*-value < 0.01 was considered as highly significant.

## CONCLUSIONS

Anti-EGFR antibody h-R3 conjugated dendriplexes as gene carriers were designed in this paper. We characterized the physicochemical properties (including DNA loading ability, particles size, zeta potential and morphology), evaluated *in vitro* toxicity, gene transfection efficacy in three cell line (EGFR-negative 293T, EGFR-expressing MCF-7 and EGFR-overexpressing HepG2), and intracellular uptake. Furthermore, we examined the *ex vivo* distribution and gene express of dendriplexes and h-R3-dendriplexes in tumor-bearing BALB/c nude mice. Dendriplexes and h-R3-dendriplexes represented excellent DNA encapsulation ability and formed unique nanostructures. Compared to dendriplexes, h-R3-dendriplexes showed lower cytotoxicity, higher gene transfection efficiency, greater cellular uptake and high nuclear accumulation in the EGFR-overexpressing HepG2 cell line. Moreover, the *ex vivo* distribution in the tumors also confirmed that h-R3-dendriplexes had much better gene delivery efficiency and higher targeted delivery than dendriplexes. Thus, the modification of h-R3 with dendriplexes on the surface contributes better efficiency to DNA delivery *in vitro* and *ex vivo*. Also, h-R3-dendriplexes for p53 delivery represented efficient cell growth inhibition and potentiated paclitaxel-induced cell death. Therefore, we believe that h-R3-dendriplex is a promising targeted gene delivery candidate, especially in EGFR-overexpressing tumor cells.
